# Trends in Social Norms Towards Smoking Between 2002 and 2015 Among Daily Smokers: Findings From the International Tobacco Control Four Country Survey (ITC 4C)

**DOI:** 10.1093/ntr/ntz179

**Published:** 2019-09-18

**Authors:** Katherine A East, Sara C Hitchman, Ann McNeill, Stuart G Ferguson, Hua-Hie Yong, K Michael Cummings, Geoffrey T Fong, Ron Borland

**Affiliations:** 1 National Addiction Centre, Institute of Psychiatry, Psychology, and Neuroscience, King’s College London, London, UK; 2 UK Centre for Tobacco and Alcohol Studies, Nottingham, UK; 3 College of Health and Medicine, University of Tasmania, Hobart, TAS, Australia; 4 Cancer Council Victoria, Melbourne, VIC, Australia; 5 School of Psychology, Deakin University, Geelong, VIC, Australia; 6 Department of Psychiatry and Behavioural Sciences, Medical University of South Carolina, Charleston, SC; 7 Department of Psychology, University of Waterloo, Waterloo, ON, Canada; 8 School of Public Health and Health Systems, University of Waterloo, Waterloo, ON, Canada; 9 Ontario Institute for Cancer Research, Toronto, ON, Canada; 10 School of Psychological Sciences, University of Melbourne, Melbourne, VIC, Australia

## Abstract

**Objective:**

To assess trends in daily smokers' social norms and opinions of smoking between 2002 and 2015 in Canada, the United States, the United Kingdom, and Australia.

**Method:**

Data were from wave 1 (2002) to wave 9 (2013–2015) of the longitudinal International Tobacco Control Four Country Survey (Canada, the United States, the United Kingdom, Australia), involving 23 831 adult daily smokers. Generalized estimating equation logistic regression models, adjusted for demographics and survey design effects, assessed associations of wave and country with outcomes: (A) over half of five closest friends smoke, (B) agreeing that people important to you believe you should not smoke, (C) agreeing that society disapproves of smoking, and (D) negative opinion of smoking.

**Results:**

Between 2002 and 2015, adjusting for covariates, (A) over half of five closest friends smoke did not change (56% vs. 55%; adjusted odds ratio [AOR] = 0.95 [95% Confidence Interval = 0.85–1.07]), (B) agreeing that people important to you believe you should not smoke generally decreased (89% vs. 82%; AOR = 0.54 [0.46–0.64]) despite an increase around 2006–2007, (C) agreeing that society disapproves of smoking increased between 2002 and 2006–2007 (83% vs. 87%; AOR = 1.38 [1.24–1.54]) then decreased until 2013–2015 (78%; AOR = 0.74 [0.63–0.88]), and (D) negative opinion of smoking decreased between 2002 and 2010–2011 (54% vs. 49%; AOR = 0.83 [0.75–0.91]) despite an increase around 2005–2006 and at the final wave (2013–2015). Except friend smoking, Canada had the greatest, and the United Kingdom the lowest, antismoking social norms and opinions.

**Conclusions:**

Except friend smoking and opinion of smoking, daily smokers' social norms became less antismoking between 2002 and 2015 despite increases around 2006–2007. Several potential explanations are discussed yet remain undetermined.

**Implications:**

Increasingly comprehensive tobacco control policies alongside decreasing smoking prevalence in Canada, the United States, the United Kingdom, and Australia have led to the assumption that smoking has become denormalized in these countries. Absent from the literature is any formal assessment of social norms towards smoking over time. Contrary to our hypotheses, this study found that the injunctive social norms of daily smokers became less antismoking between 2002 and 2015, despite increases around 2006–2007. There was no change over time in the proportion of daily smokers who report that over half of their five closest friends smoke.

## Introduction

Social norms have an important impact on human behavior,^1–4^ and can be categorized into two distinct domains. Descriptive norms refer to a person's perceptions of how others behave (eg, how common smoking is), while injunctive norms refer to a person's perceptions of how others think people ought to behave (eg, approval of smoking).^2–4^ By extension, denormalization is the process of changing a person's perceptions about a behavior from more common and/or approved to less common and/or approved; renormalization is the reverse.

Social norms towards tobacco smoking can be important sources of influence for smoking intentions, uptake, and cessation.^5–9^ Several tobacco control efforts focus on the denormalization of smoking and, in conceptual models, normalization beliefs are often placed on the causal pathway between tobacco control policies and behavioral outcomes.^10,11^ Many efforts have been found to reduce smoking prevalence and promote cessation,^12–15^ and smoking prevalence has decreased alongside increasingly comprehensive tobacco control policies.^16^ Smoking is thus theorized to have become increasingly denormalized in many Western countries. Indeed, a recent study among British youth found that both prevalence of smoking and prevalence of perceiving that smoking is OK have decreased between 1998 and 2015.^17^ However, there is no research of which we are aware assessing both descriptive and injunctive norms towards smoking over time.

**Figure 1.**
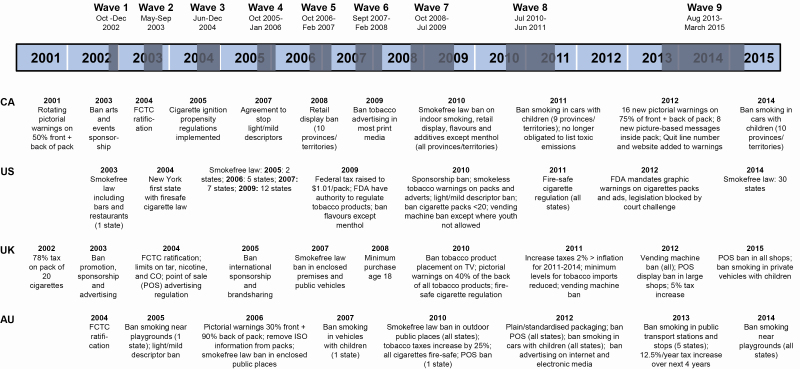
Timeline of the ITC 4C and tobacco control policies in Canada (CA), the United States (US), the United Kingdom (UK), and Australia (AU) between 2001 and 2015.^20^

This paper uses data from the four countries of the International Tobacco Control 4 Country Survey (ITC 4C), Canada, the United States, the United Kingdom, and Australia, collected between 2002 and 2015, to assess trends in social norms over time. This century there have been increasingly comprehensive tobacco control policies in Canada, the United States, the United Kingdom, and Australia (Figure 1). While there are similarities between these countries, there are important differences, with the United States generally lagging in the implementation of several tobacco control initiatives, including updated health warnings, retail cigarette marketing restrictions, and nationwide smoke-free policy (Figure 1). Since 2002, the United Kingdom has had the highest, and Australia the lowest, prevalence of tobacco smoking,^18^ and in all four countries prevalence has decreased.^18^ Kasza et al.^19^ assessed reasons smokers think about quitting between 2002 and 2015 in these four countries and found an upward trend in societal disapproval of smoking as a reported reason for quitting in the United States, a downward trend in Canada, and non-linear trends in the United Kingdom and Australia, suggesting possible differential trends between countries.

This study uses daily smokers from the longitudinal ITC 4C (Canada, the United States, the United Kingdom, Australia) to assess trends between 2002 and 2015 in one descriptive norm (A) reporting that over half of your five closest friends smoke; two injunctive norms, agreeing that (B) people important to you believe you should not smoke, and (C) society disapproves of smoking; and (D) respondents' overall opinion of smoking. Given increased smoking restrictions and decreased prevalence, we hypothesized that all four measures would indicate denormalization of smoking over time, although the Kasza et al.^19^ study suggests possibly more complex relationships. Between-country differences are also explored.

## Methods

### Pre-registration

The analysis plan was pre-registered on the Open Science Framework.^21^

### Sample

This study used data from wave 1 (2002) to wave 9 (2013–2015) of the longitudinal ITC 4C in Canada, the United States, the United Kingdom, and Australia. Details about the sampling and design are described elsewhere.^22–24^ Briefly, nationally representative samples of ~2000 current smokers (≥100 cigarettes in lifetime and ≥1 cigarette in the past 30 days) age 18+ were recruited from each country. All respondents were re-contacted annually, and new smokers were recruited to offset attrition. Waves 1–6 used Computer-Assisted Telephone Interviewing. Waves 7–9 used Computer-Assisted Telephone Interviewing and internet surveys. Wave-to-wave recruitment response rates (for newly recruited respondents) ranged from 13% (the United Kingdom, wave 5) to 50% (Canada, wave 3); wave-to-wave follow-up rates (for re-contacted respondents), ranged from 66% (the United Kingdom, wave 5) to 91% (Australia, wave 3).^22,23^ Prior analyses have found good correspondence between the demographic profiles of ITC 4C respondents and national benchmark surveys.^22–24^ This study included only daily smokers at each wave (23 831 respondents, 57 086 observations). Respondents who quit and relapsed to daily smoking were added back into the sample when they relapsed.

### Measures

#### Outcome Variables

##### (A) Over Half of Five Closest Friends Smoke

“Of the five closest friends or acquaintances that you spend time with on a regular basis, how many of them are smokers? 0–5”. For analyses, responses were dichotomized as under half (0–2) versus over half (3–5), similar to previous ITC studies.^25^ Responses were dichotomized because the assumptions of (1) normality of residuals and homoscedasticity for linear regression, and (2) proportional odds for ordinal logistic regression were violated.^21^

##### (B) People Important to You Believe You Should not Smoke

“People who are important to you believe that you should not smoke. Strongly Agree, Agree, Neither Agree nor Disagree, Disagree, Strongly Disagree”. For analyses, responses were dichotomized as agree (Strongly Agree, Agree) versus not agree (Neither Agree nor Disagree, Disagree, Strongly Disagree).

##### (C) Society Disapproves of Smoking

“Society disapproves of smoking. Strongly Agree, Agree, Neither Agree nor Disagree, Disagree, Strongly Disagree”. For analyses, responses were dichotomized as agree (Strongly Agree, Agree) versus not agree (Neither Agree nor Disagree, Disagree, Strongly Disagree).

##### (D) Opinion of Smoking

“What is your overall opinion of smoking? Is it...? Very Positive, Positive, Neither Positive nor Negative, Negative, Very Negative”. For analyses, responses were dichotomized as negative (Negative, Very Negative) versus not negative (Very Positive, Positive, Neither Positive nor Negative). Coding deviated from the pre-registration^21^ because 11.2% of respondents answered “Very positive” or “Positive”. Therefore, negative opinion was modeled as the majority response.

#### Predictor Variables

Survey wave: 1 (collected in 2002), 2 (2003), 3 (2004), 4 (2005–2006), 5 (2006–2007), 6 (2007–2008), 7 (2008–2009), 8 (2010–2011), and 9 (2013–2015) (Figure 1).

Country: Canada, the United States, the United Kingdom, Australia.

#### Covariates

Covariates measured at baseline: age (18–24, 25–39, 40–54, 55+), gender (male, female), ethnicity (majority, minority).

Covariates measured at each wave: Annual household income (low, moderate, high, no answer), education (low, moderate, high, no answer), Heaviness of Smoking Index (HSI)^26^ (0–6, 6 = greater dependence), survey mode (telephone, internet), time-in-sample (1–9 waves; number of waves respondent was involved in), and time-between-waves (0–3.5 years; time since respondent last completed a survey). Time-in-sample was included to control for potential participation effects; ^24,27^ time-between-waves was included because the time between survey waves differed by country towards the end of the study and we wanted to control for these differences. The questionnaires^28^ and details on coding of income, education, and ethnicity^19,21^ are available elsewhere.

### Analyses

Four logistic regression models were estimated using Generalized Estimating Equations (GEE) to assess associations between each outcome (A)–(D) and country and wave, adjusting for covariates. Average probabilities of each social norm (A)–(D) were predicted from these models using Stata's margins command and plotted; this differed from the pre-registration because the results were influenced by time-in-sample.^21^ GEE account for correlations in the longitudinal data. Correlations between observations from the same individuals were modeled specifying an unstructured within-person correlation matrix.

First, wave was first treated as categorical to aid interpretation of nonlinear trends. Second, wave was treated as continuous and linear, quadratic, and cubic trends were assessed for (A)–(D) using hierarchical logistic regression (model 1 (linear): wave + country + covariates; model 2 (quadratic): wave^2^ + wave + country + covariates; model 3 (cubic): wave^3^ + wave^2^ + wave + country + covariates); the highest-order significant (*p* < .05) model was reported for each of (A)–(D). Third, wave (categorical) by country interactions were assessed for (A)–(D); where interactions were observed (*p* < .05), average predicted probabilities were plotted using Stata's margins command and compared pairwise using 99% confidence intervals due to multiple comparisons. Linear, quadratic, and cubic trends, and interactions, deviated from the pre-registration following observation of linear and nonlinear trends.^21^

Country was treated as a time-invariant, and wave as a time-variant, predictor. Age, gender, and ethnicity were treated as time-invariant covariates. Income, education, HSI, survey mode, time-in-sample, and time-between-waves were treated as time-variant covariates. Only the results for wave and country are reported below.

Analyses were run using Stata v15.^29^ All data were weighted. Weights were calculated using estimated population values from national benchmark surveys incorporating gender, age, and region.^22–24^

#### Missing Data

For outcomes (A)–(D), all “Not applicable”, “Refused”, and “Don't know” responses were coded as missing and multiple imputation was used for these values: friend smoking (*n* = 2057, 3.54%, observations imputed), people important to you believe you should not smoke (*n* = 1626, 2.79%), society disapproves of smoking (*n* = 1822, 3.13%), opinion of smoking (*n* = 2113, 3.63%). Multiple imputation was also used for missing data on income (*n* = 1153, 1.98%) and HSI (*n* = 1994, 3.43%), which deviated from the pre-registration due to unanticipated missing data on these two covariates.^21^ There were no missing data on any other variables. Data were not Missing Completely At Random, because wave, age, gender, ethnicity, education, mode, time-in-sample, and time-between-waves were associated with missingness (*p* < .05). Data were therefore assumed to be Missing At Random. Missing values were imputed using chained equations. One model was used specifying imputation via multinomial logistic regression for all social norms measures, income, and HSI, with country, age, gender, ethnicity, education, survey mode, time-in-sample, time-between-waves, wave (categorical, linear, quadratic, cubic), and wave (linear, quadratic, cubic) by country interactions as predictors. Survey weights were included. Twenty imputed datasets were created because <30% of data were missing.^30^ Multiple imputation results in valid statistical inferences that reflect uncertainty due to missing values while enabling sample size to be maximized.^31^

#### Sensitivity Analyses

The following sensitivity analyses were conducted to identify potential problems with estimation: (1) prevalence estimates and logistic regression GEE with multiple imputation versus complete case analyses, (2) logistic regression GEE specifying an unstructured versus exchangeable correlation matrix, (3) prevalence estimates for the re-contacted versus newly recruited samples (additional to pre-registration).^21^ The interpretation of the results remained unchanged during all sensitivity analyses.

## Results

### Sample Characteristics

Of the 23 831 respondents, 5545 were from Canada, 7832 from the United States, 5421 from the United Kingdom, and 5033 from Australia. The modal demographic categories were 25–54 years, male, majority ethnicity, and moderate household income and education (Table 1). Most were recruited in wave 1 and took part in only one survey wave (Table 1). Supplementary Table 1 has further details on the sample characteristics at each wave.

**Table 1.** Sample Characteristics at Wave of Recruitment for Each Respondent

%	Canada	United States	United Kingdom	Australia	Overall
	*n* = 5545	*n* = 7832	*n* = 5421	*n* = 5033	*n* = 23 831
Age (y)					
18–24	12.46	13.52	13.14	14.23	13.34
25–39	32.04	29.97	32.70	35.52	32.23
40–54	35.37	34.98	29.93	32.84	33.47
55+	20.14	21.53	24.24	17.41	20.96
Gender: female	46.21	46.33	49.10	45.43	46.75
Ethnicity: majority	88.84	78.84	94.17	87.51	86.47
Annual household income					
Low	25.80	37.63	28.76	25.51	30.33
Moderate	32.95	32.31	32.16	32.95	32.98
High	30.83	24.50	29.62	34.74	29.28
No answer	8.58	5.57	9.46	6.79	7.41
Education					
Low	49.39	47.66	58.73	64.46	54.08
Moderate	35.81	38.07	26.95	22.23	31.71
High	14.37	14.17	13.55	12.97	13.83
No answer	0.43	0.10	0.76	0.34	0.38
HSI^a^ (mean (SE^b^))	2.89 (0.02)	2.84 (0.02)	2.66 (0.02)	2.95 (0.03)	2.83 (0.01)
Wave of recruitment					
Wave 1	37.07	25.09	41.67	42.57	35.30
Wave 2	8.59	8.10	4.11	4.63	6.58
Wave 3	9.16	10.65	10.38	9.78	10.06
Wave 4	8.77	8.81	8.95	6.77	8.41
Wave 5	10.13	9.04	10.42	12.80	10.39
Wave 6	9.26	8.53	9.11	9.98	9.13
Wave 7	5.61	4.46	6.25	2.24	4.67
Wave 8^c^	3.45	4.32	0.00	3.84	3.03
Wave 9	7.98	21.00	9.12	7.40	12.42
Time-in-sample					
1 wave	39.06	52.40	39.34	35.97	41.90
2 waves	22.10	20.61	22.83	22.61	22.01
3 waves	14.05	11.10	14.55	14.60	13.52
4 waves	9.45	6.89	9.53	10.51	9.05
5 waves	5.95	4.03	5.95	6.48	5.57
6 waves	4.08	2.38	3.57	4.20	3.54
7 waves	2.71	1.39	2.29	2.91	2.31
8 waves	1.71	0.82	1.25	1.73	1.37
9 waves	0.89	0.38	0.68	1.00	0.73

Data are weighted.

^a^HSI = Heaviness of Smoking Index.

^b^SE = standard error.

^c^There was no replenishment at wave 8 in the United Kingdom due to resource constraints.

### Over Half of Five Closest Friends Smoke

#### Trend

**Figure 2.**
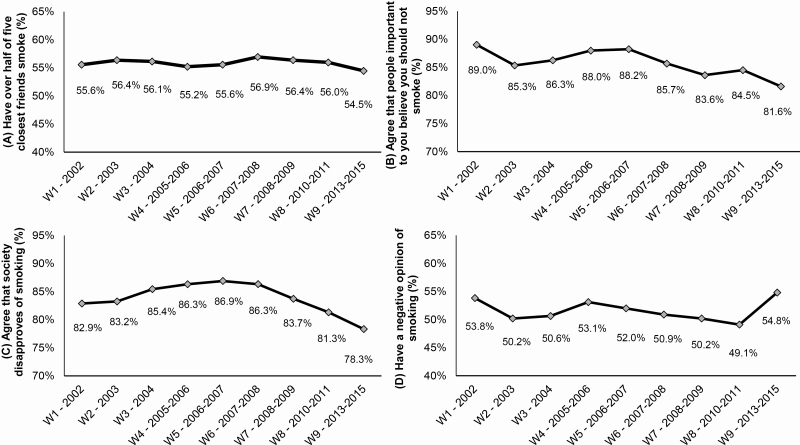
Average predicted probabilities of (A) having over half of five closest friends smoke, (B) agreeing that people important to you believe you should not smoke, (C) agreeing that society disapproves of smoking, and (D) having a negative opinion of smoking, by wave (*N* = 57 086 observations from 23 831 respondents). Data are from binary logistic regression analyses, adjusted for country, age, gender, ethnicity, income, education, heaviness of smoking, survey mode, time in sample, time-between-waves, and weighted. W = wave.

At wave 1 (2002), 55.6% (adjusted for covariates) reported that over half of their five closest friends smoke and this showed little change over time (Figure 2A; Table 2, A). There was also little evidence of any linear, quadratic, or cubic trends (Table 2, A).

**Table 2.** Adjusted Associations Between Wave and Country and (A) Having Over Half of Five Closest Friends Smoke, (B) Agreeing That People Important to You Believe You Should not Smoke, (C) Agreeing That Society Disapproves of Smoking, and (D) Having a Negative Opinion of Smoking, Among Daily Smokers (*N* = 57 086 Observations From 23 831 Respondents)

	(A) Over half of five closest friends smoke		(B) Agree that people important to you believe you should not smoke		(C) Agree that society disapproves of smoking		(D) Negative opinion of smoking	
	AOR (95% CI)	*p*	AOR (95% CI)	*p*	AOR (95% CI)	*p*	AOR (95% CI)	*p*
Wave								
Categorical								
1 - 2002 (ref.)	1.00		1.00		1.00		1.00	
2 - 2003	1.04 (0.98–1.10)	.250	**0.71 (0.65–0.78)**	**<.001**	1.03 (0.94–1.12)	.557	**0.86 (0.81–0.91)**	**<.001**
3 - 2004	1.03 (0.96–1.10)	.477	**0.77 (0.69–0.86)**	**<.001**	**1.22 (1.11–1.35)**	**<.001**	**0.88 (0.82–0.94)**	**<.001**
4 - 2005–2006	0.98 (0.92–1.06)	.673	0.90 (0.81–1.01)	.072	**1.31 (1.18–1.47)**	**<.001**	0.97 (0.91–1.04)	.423
5 - 2006–2007	1.00 (0.93–1.08)	.998	0.92 (0.82–1.04)	.178	**1.38 (1.24–1.55)**	**<.001**	**0.93 (0.86–1.00)**	**.045**
6 - 2007–2008	1.06 (0.98–1.15)	.137	**0.73 (0.65–0.83)**	**<.001**	**1.32 (1.17–1.48)**	**<.001**	**0.89 (0.82–0.96)**	**.002**
7 - 2008–2009	1.04 (0.95–1.13)	.433	**0.62 (0.55–0.71)**	**<.001**	1.06 (0.94–1.20)	.314	**0.86 (0.79–0.94)**	**.001**
8 - 2010–2011	1.02 (0.92–1.13)	.747	**0.67 (0.57–0.78)**	**<.001**	0.89 (0.77–1.03)	.130	**0.83 (0.75–0.91)**	**<.001**
9 - 2013–2015	0.95 (0.85–1.07)	.436	**0.54 (0.46–0.64)**	**<.001**	**0.74 (0.63–0.87)**	**<.001**	1.04 (0.93–1.17)	.503
Linear (wave)	1.00 (0.99–1.01)	.971	**0.77 (0.67–0.89)**	**<.001**	**1.28 (1.22–1.34)**	**<.001**	**0.95 (0.92–0.99)**	**.005**
Quadratic (wave^2^)	1.00 (0.995–1.01)	.395	**1.06 (1.03–1.10)**	**.001**	**0.97 (0.97–0.98)**	**<.001**	**1.00 (1.001–1.01)**	**.008**
Cubic (wave^3^)	1.00 (0.998–1.01)	.425	**0.995 (0.99–0.998)**	**<.001**	1.00 (0.996–1.000)	.115	1.00 (0.999–1.002)	.603
Country								
Canada (ref.)	1.00		1.00		1.00		1.00	
United States	**1.10 (1.03–1.19)**	**.007**	0.99 (0.89–1.10)	.822	**0.63 (0.57–0.69)**	**<.001**	**0.71 (0.66–0.77)**	**<.001**
United Kingdom	1.03 (0.96–1.11)	.453	**0.58 (0.52–0.64)**	**<.001**	**0.59 (0.54–0.65)**	**<.001**	**0.66 (0.62–0.71)**	**<.001**
Australia	0.94 (0.87–1.02)	.131	**0.84 (0.75–0.94)**	**.003**	**0.74 (0.67–0.82)**	**<.001**	**0.88 (0.81–0.95)**	**.001**

AOR = adjusted odds ratio, adjusted for age, gender, ethnicity, income, education, HSI = Heaviness of Smoking Index, survey mode, time-in-sample, time-between-waves. 95% CI = 95% confidence interval. 95% CIs are reported to three decimal places where they are close to 1.00 (±0.005). For linear, quadratic, and cubic wave terms, the results are reported from the highest-order significant (*p* < .05) model (cubic for outcome (ii) [wave, wave^2,^ wave^3^ all from model 3; see Analyses section], quadratic for outcomes (iii)–(iv) [wave, wave^2^ both from model 2; see Analyses section]); where models are not significant (all models for outcome (i), cubic models for outcomes (iii)–(iv)) the results are reported from the corresponding wave term of that model (ie, wave from model 1, wave^2^ from model 2, wave^3^ from model 3; see Analyses section). The full models including all covariates are shown in Supplementary Table 3.

Bold values represent statistical significance at *p* = .05.

There was a declining trend in unadjusted prevalence at each wave (Supplementary Table 2) but this was found to be an artifact of time-in-sample: in unadjusted GEE logistic regression, the odds of having over half of friends smoke decreased between wave 2 (2003) and wave 9 (2013–2015) (all OR < 1.00, *p* < .05; data not shown), yet when time-in-sample was added this association was attenuated (all *p* > .05; data not shown). As time-in-sample increased the odds of having over half of friends smoke decreased (Supplementary Table 3).

We also explored whether reporting “at least one of five closest friends smoke” (0 vs. 1–5) changed over time, for comparison (additional to pre-registration).^21^ Except a slight increase from 87.6% (adjusted for covariates) at wave 1 (2002) to 89.0% at wave 5 (2006–2007; adjusted odds ratio = 1.15 [1.03–1.29], *p* = .014), there was little change over time in reporting that at least one of five closest friends smoke (all *p* > .05; data not shown).

#### Between-Country Differences

Respondents from the United States had greater odds of having over half of their five closest friends smoke compared with Canada and Australia (Table 2, A).

#### Wave-by-Country Interactions

There was a wave-by-country interaction (*F*(24,1200000) = 1.73, *p* = .015), such that there was no change over time in Canada, the United States or Australia, but in the United Kingdom, compared with wave 2 (2003), friend smoking was lower at wave 4 (2005–2006) and wave 9 (2013–2015) (Supplementary Figure 1, left panels).

### People Important to You Believe You Should Not Smoke

#### Trend

At wave 1 (2002), 89.0% (adjusted for covariates) agreed that people important to you believe you should not smoke (Figure 2B). This decreased to 85.3% at wave 2 (2003), increased gradually to 88.2% at wave 5 (2006–2007) and subsequently decreased again to 81.6% at wave 9 (2013–2015) (Figure 2B; Table 2, B). Trends analyses indicated a cubic trend (Table 2, B; with *p* ≤ .001 for all adjusted odds ratios for the linear, quadratic, and cubic wave terms) reflecting the decrease, increase, and decrease again in agreement that people important to you believe you should not smoke. These results were similar to the unadjusted prevalence at each wave (Supplementary Table 2).

#### Between-Country Differences

Respondents from Canada had greater odds of agreeing that people important to you believe you should not smoke compared with the United Kingdom and Australia, and those from Australia and the United States had greater odds of agreeing that people important to you believe you should not smoke compared with the United Kingdom (Table 2, B).

#### Wave-by-Country Interactions

There was little evidence of a wave-by-country interaction (*F*(24,3200000) = 1.41, *p* = .086), suggesting the association between wave and agreeing that people important to you believe you should not smoke did not differ by country.

### Society Disapproves of Smoking

#### Trend

At wave 1 (2002), 82.9% (adjusted for covariates) agreed that society disapproves of smoking (Figure 2C). This increased to 86.9% at wave 5 (2006–2007), then decreased to 78.3% until wave 9 (2013–2015) (Figure 2C; Table 2, C). Trends analyses indicated a quadratic trend (Table 2, C; with *p* < .001 for both adjusted odds ratios for the linear and quadratic wave terms), reflecting the increase and subsequent decrease in agreement that society disapproves of smoking. These results were similar to the unadjusted prevalence at each wave (Supplementary Table 2).

#### Between-Country Differences

Respondents from Canada had greater odds of agreeing that society disapproves of smoking compared with the United States, the United Kingdom, and Australia, and those from Australia had greater odds than the United Kingdom (Table 2, C).

#### Wave-by-Country Interactions

There was a wave-by-country interaction (*F*(24,1500000) = 1.77, *p* = .011), such that in the United States, the United Kingdom, and Australia agreeing that society disapproves of smoking increased between wave 1 (2002) and wave 5 (2006–2007) then decreased between wave 5 (2006–2007) and wave 9 (2013–2015), whereas in Canada there was no change between wave 1 (2002) and wave 6 (2007–2008) yet a decrease between wave 6 (2007–2008) and wave 9 (2013–2015) (Supplementary Figure 1, center panels).

### Negative Opinion of Smoking

#### Trend

At wave 1 (2002), 53.8% (adjusted for covariates) had a negative opinion of smoking (Figure 2D). This decreased to 50.2% at wave 2 (2003), increased gradually to 53.1% at wave 4 (2005–2006), decreased gradually again to 49.1% at wave 8 (2010–2011) and sharply increased again to 54.8% at wave 9 (2013–2015) (Figure 2D; Table 2, D). Trends analyses indicated a quadratic trend (Table 2, D; with *p* ≤ .001 for both adjusted odds ratios for the linear and quadratic wave terms), reflecting the overall linear downwards trend with a slight increase between waves 2–4 and some recovery of negative opinion at the final wave. Except wave 9, these results were similar to the unadjusted prevalence at each wave (Supplementary Table 2).

#### Between-Country Differences

Respondents from Canada had greater odds of having a negative opinion of smoking compared with the United States, the United Kingdom, and Australia (Table 2, D). Those from Australia had greater odds of having a negative opinion of smoking compared with the United States and the United Kingdom (Table 2, D).

#### Wave-by-Country Interactions

There was a wave-by-country interaction (*F*(24,828753.4) = 1.77, *p* = .011), such that in Canada negative opinion of smoking decreased between wave 1 (2002) and wave 8 (2010–2011), yet increased between wave 8 (2010–2011) and wave 9 (2013–2015), while in the United Kingdom negative opinion increased between wave 2 (2003) and wave 4 (2005–2006) then remained unchanged (Supplementary Figure 1, right panels). There was no change over time in the United States or Australia (Supplementary Figure 1, right panels).

## Discussion

We hypothesized that all four measures used in this study would indicate denormalization of smoking between 2002 and 2015. Contrary to our hypotheses: the descriptive norm (A) reporting that over half of five closest friends smoke did not change after adjusting for covariates; the two injunctive norms, agreeing that (B) people important to you believe you should not smoke, and (C) society disapproves of smoking, generally decreased between 2002 and 2013–2015 despite increases around 2006–2007; (D) negative opinion of smoking generally decreased between 2002 and 2010–2011 despite an increase around 2005–2006 and some recovery at the final wave (2013–2015). Trends were similar across the four countries, and Canada had the greatest, and the United Kingdom the lowest, antismoking injunctive norms and opinions.

This study finds that there has been a shift towards less antismoking injunctive norms among daily smokers, beginning around 2006/2007. These results complement a previous study using the same ITC 4C dataset,^19^ which assessed societal disapproval of smoking as a reported reason for quitting and failed to find any clear increases from 2002–2015, except in the United States. There are several speculated reasons for these trends, discussed below.

The first explanation for the shift towards less antismoking injunctive norms among daily smokers from around 2006/2007 is smoke-free legislation. By 2006/2007, smoke-free policies were fully implemented in the United Kingdom and Australia, in seven of ten Canadian provinces, and in thousands of local communities and seven states covering most of the US population (Figure 1). Because rules limiting smoking in public had become commonplace and accepted by smokers and nonsmokers, and because reduced opportunities to smoke might have led smokers to reduce their cigarette consumption,^27^ smokers may perceive less disapproval from those around them. However, this proposition would be difficult to assess.

Second, the introduction of social media platforms, such as Twitter, Facebook, and YouTube, around 2005/2006 may have contributed towards less antismoking injunctive norms and changing opinions. Pro-smoking online content is widely available,^32^ and previous research has indicated associations with social norms and attitudes towards smoking.^33^ Moreover, the internet might give rise to extremist bloggers or a more widespread distrust in the government and public health experts, which could be linked to normalization beliefs. Therefore, social media may challenge antismoking norms and/or perpetuate existing pro-smoking norms; this may have magnified over time with increasingly widespread and sophisticated platforms.

There are also some explanations for the general downwards trends in injunctive norms and negative opinion of smoking that are unlikely, but some may think plausible. There are debated concerns that the introduction of electronic cigarettes (e-cigarettes) in the mid-2000s, and the increase in their marketing and use, could “renormalize” smoking.^34–37^ However, e-cigarettes only became popular since around 2010,^38^ yet the shift in norms and opinions was seen from 2006/2007. Policies such as mass media campaigns, sponsorship and advertising bans, and taxation (Figure 1) also constitute unlikely explanations, since it is difficult to see any clear pattern corresponding with the trends observed. Perhaps a more plausible explanation is that lower smoking prevalence and numerous tobacco control policies may have led to a presumption that smoking is no longer a public health priority, given obesity, dementia, opioids, and other competing health concerns. However, these explanations cannot account for the increases around 2006/2007, or the trend in opinion of smoking.

Finally, it is possible that smoking is no longer seen as a societal problem due to increasing disparities in smoking prevalence between advantaged and disadvantaged groups in the four countries. Daily smokers are more likely to be of lower socioeconomic status,^39–41^ older,^41^ and have weaker antismoking norms.^5^ Therefore, the individuals in this study may represent a group who are increasingly marginalized, being aware of the dangers of smoking but lacking the resources to quit. Such individuals may hold more entrenched or polarized social norms and opinions, or adopt a siege mentality, which may be increasingly pronounced with decreasing smoking prevalence and wide-scale adoption of tobacco control policies. Tobacco has also become less affordable over time, particularly among those of lower socioeconomic status,^42^ leading to further disparities and perhaps further resentment, particularly among disadvantaged groups. However, as above, these explanations cannot account for the increases around 2006/2007, unless any marginalization effects were amplified by smoke-free policy, and although our sample did become older over time they also became better educated and less heavy smokers (Supplementary Table 1), counter to the idea of increased marginalization.

The implications of changing social norms and opinions of daily smokers are unclear. Descriptive and injunctive norms have been associated with smoking behaviors and intentions.^5–9,43^ However, adult smoking prevalence has decreased from 2002 to 2015 alongside increasingly comprehensive policies in all four ITC 4C countries.^18^ This may question whether smoking denormalization is a valid approach to tobacco control, or at least that smoker’s social norms may be less related to smoking policies and prevalence rates than theorized; ^10,11,44^ this reflects findings for injunctive norms from a recent ITC study in Europe.^25^ Despite this, Kasza et al.^19^ found that reporting societal disapproval as a reason to quit smoking increased the odds of making a quit attempt. It is, therefore, possible that the observed trends could preempt an attenuation of declines in smoking. In the literature, concerns about smoking “renormalization” focus on e-cigarettes promoting youth smoking uptake; ^34,37,45^ it is important to not generalize such arguments to the changing social norms of daily adult smokers seen in this study, especially given evidence that e-cigarettes may help some smokers quit.^46^ Moreover, a recent study among British youth found that prevalence of perceiving smoking as OK decreased from 1998 to 2015,^17^ contrary to our findings; trends in norms and opinions may thus differ across different groups. Ongoing longitudinal surveys, such as the ITC Surveys and US Population Assessment of Tobacco and Health Surveys, are critical to continue monitoring norms and extend our findings among smokers, nonsmokers, and those who quit smoking.

Consistent with previous studies,^5^ overall, smokers in Canada had the greatest, and the United Kingdom the lowest, antismoking injunctive norms and negative opinion of smoking. These differences could be explained by a longer history of antismoking policies in Canada compared with the United Kingdom.^5^ Both trends and country differences in having over half of five closest friends who smoke showed little obvious relation to smoking policies (Figure 1) or prevalence rates.^18^ This is in contrast to a recent cross-sectional ITC study among smokers, which did find that friend smoking was higher in European countries with greater smoking prevalence.^25^ While there are some differences between the four ITC 4C countries, their policies are all antismoking to a large degree (Figure 1). There may be greater contrast in levels of social norms and trends over time in low- and middle-income countries at earlier stages of the tobacco epidemic.

It is important to consider this study's limitations. First, the sample involved adult daily smokers who, as stated above, represent a unique group. The results, therefore, cannot be generalized to the social norms or opinions of non-daily smokers, quitters, never-smokers, or youth; replication is required using surveys of the general population in each country. Second, although the adjusted odds of having over half of five closest friends smoke did not change over time, odds of having *at least one* slightly increased at 2006–2007. Average number of smoking friends may have shown further changes but could not be assessed due to violation of linear and ordinal logistic regression assumptions. Third, there is some lack of clarity as to what these social norms measures mean. For example, following the implementation of smoke-free policies, daily smokers might perceive less societal disapproval because they experience fewer negative reactions, rather than due to any true change in societal disapproval. More nuanced measures of social norms, assessed among both smokers and nonsmokers, may aid interpretation of findings. Fourth, most participants only took part in one survey wave which may have impacted the findings, particularly because time-in-sample had an effect on friend smoking and perceived societal disapproval of smoking; however, we adjusted for time-in-sample and sensitivity analyses indicated similar results for the re-contacted and newly recruited samples.

Strengths include large sample size, weighted and nationally representative data, adjustment for demographics associated with social norms,^5^ and adjustment for survey characteristics which may influence responses. Further, this study was the first to our knowledge to longitudinally and consistently assess social norms and opinions of smoking over time and across countries and has raised important issues regarding how they might be changing.

To conclude, injunctive social norms of daily smokers have generally become less antismoking between 2002 and 2015, despite increases around 2006/2007. There was no change in reporting that over half of five closest friends smoke. Trends were similar across the four countries, although there were overall differences with Canada generally having the greatest, and the United Kingdom the lowest, antismoking injunctive norms and opinions. While country differences might be explained to an extent by different tobacco control policies, common trends were contrary to our hypotheses and so the proposed explanations should be considered tentative until further research identifies which, if any, may be implicated.

## Funding

The International Tobacco Control Four Country Survey has been funded by the US National Cancer Institute (P50 CA111326, P01 CA138389, R01 CA100362, R01 CA090955), Canadian Institutes of Health Research (MOP-57897, MOP-79551, MOP-115016, and FDN-148477), Commonwealth Department of Health and Aging, National Health and Medical Research Council of Australia (265903, 450110, 1005922, and 1106451), Cancer Research UK (C312/A3726, C312/A6465, C312/A11039, C312/A11943), Robert Wood Johnson Foundation (045734), and Canadian Tobacco Control Research Initiative (014578). Additional support was provided to GTF from a Senior Investigator Award from the Ontario Institute for Cancer Research and a Prevention Scientist Award from the Canadian Cancer Society Research Institute. KE’s PhD is funded by the UK Centre for Tobacco and Alcohol Studies (MR/K023195/1), and KE received additional support to work on this study from the Society for the Study of Addiction (SSA).

## Declaration of Interests

*SF has consulted for GlaxoSmithKline Consumer Healthcare and Chrono Therapeutics on matters relating to smoking cessation and has received researcher-initiated project grant funding from Pfizer (through the GRAND initiative), and also serves on an advisory board for Johnson & Johnson. KMC has received grant funding from Pfizer, Inc. to study the impact of a hospital-based tobacco cessation intervention and also has served as an expert witness in litigation filed against the tobacco industry. GTF has served as an expert witness on behalf of governments in litigation involving the cigarette industry. All other authors have no conflicts of interest to declare.*

## Supplementary Material

ntz179_suppl_Supplementary_Material
