# Supplementary material for: Trends in Social Norms Towards Smoking Between 2002 and 2015 Among Daily Smokers: Findings From the International Tobacco Control Four Country Survey (ITC 4C)
Source: Nicotine Tob Res. 2019 Sep 18;23(1):203–11. doi: 10.1093/ntr/ntz179 (PMC7789950; doi:10.1093/ntr/ntz179)
Supplement: ntz179_suppl_Supplementary_Material [file ntz179_suppl_supplementary_material.docx]

**Supplementary Table 1.** Unadjusted cross-sectional prevalence of all covariates, by Wave (N=57,086 observations from 23,831 respondents). Data are weighted. W=Wave.

|  | **W1 2002** | **W2**  **2003** | **W3**  **2004** | **W4**  **2005-2006** | **W5**  **2006-2007** | **W6**  **2007-2008** | **W7**  **2008-2009** | **W8**  **2010-2011** | **W9**  **2013-2015** |
| --- | --- | --- | --- | --- | --- | --- | --- | --- | --- |
| **Age** (%) |  |  |  |  |  |  |  |  |  |
| 18-24 | 14.82 | 12.34 | 11.34 | 10.19 | 9.10 | 8.51 | 7.33 | 6.29 | 7.86 |
| 25-39 | 32.65 | 30.84 | 29.52 | 29.35 | 29.90 | 28.94 | 27.38 | 25.59 | 27.12 |
| 40-54 | 33.34 | 35.77 | 37.32 | 38.00 | 38.34 | 38.78 | 39.97 | 40.89 | 37.47 |
| 55-max | 19.19 | 21.06 | 21.82 | 22.46 | 22.66 | 23.77 | 25.31 | 27.22 | 27.55 |
| **Gender: Female** (%) | 46.62 | 47.53 | 48.67 | 48.63 | 49.00 | 48.65 | 48.19 | 48.64 | 47.16 |
| **Ethnicity: Majority** (%) | 87.03 | 87.83 | 88.78 | 89.14 | 88.86 | 89.16 | 89.95 | 90.03 | 87.28 |
| **Income** (%) |  |  |  |  |  |  |  |  |  |
| Low | 29.53 | 29.52 | 29.19 | 30.03 | 29.93 | 27.33 | 27.04 | 27.62 | 33.21 |
| Moderate | 35.39 | 34.85 | 35.72 | 34.37 | 33.30 | 33.46 | 31.68 | 31.35 | 29.98 |
| High | 27.33 | 28.24 | 28.72 | 29.17 | 29.81 | 32.24 | 33.31 | 32.77 | 30.74 |
| No answer | 7.75 | 7.38 | 6.37 | 6.42 | 6.96 | 6.97 | 7.97 | 8.27 | 6.07 |
| **Education** (%) |  |  |  |  |  |  |  |  |  |
| Low | 57.46 | 54.91 | 54.72 | 54.20 | 54.64 | 52.21 | 51.87 | 50.24 | 46.35 |
| Moderate | 31.21 | 33.79 | 32.31 | 30.89 | 30.36 | 31.88 | 31.39 | 32.57 | 34.84 |
| High | 10.90 | 10.99 | 12.55 | 14.56 | 14.71 | 15.70 | 16.47 | 16.85 | 18.24 |
| No answer | 0.43 | 0.32 | 0.42 | 0.35 | 0.29 | 0.22 | 0.27 | 0.34 | 0.57 |
| **HSI** (mean (SE)) | 2.89 (0.02) | 2.76 (0.02) | 2.81 (0.02) | 2.73 (0.02) | 2.82 (0.02) | 2.83 (0.02) | 2.82 (0.02) | 2.94 (0.03) | 2.65 (0.03) |
| **Survey mode: Internet** (%) | 0.00* | 0.00* | 0.00* | 0.00* | 0.00* | 0.00* | 2.12 | 29.84 | 67.10 |
| **Time-in-sample** (mean (SE)) | 1.00 (0.00) | 1.78 (0.01) | 2.18 (0.01) | 2.60 (0.02) | 2.69 (0.02) | 2.90 (0.03) | 3.42 (0.03) | 4.04 (0.04) | 2.86 (0.04) |
| **Time-between-Waves**  (mean (SE)) | 0.00 (0.00) | 0.44 (0.00) | 0.70 (0.01) | 0.88 (0.01) | 0.61 (0.01) | 0.61 (0.01) | 0.85 (0.01) | 1.36 (0.01) | 1.18 (0.02) |

HSI=Heaviness of Smoking Index. SE=standard error. *Internet surveys were only introduced from Wave-7 (2008-2009).

**Supplementary Table 2.** Unadjusted cross-sectional prevalence of (A) having over half of five closest friends smoke, (B) agreeing that people important to you believe you should not smoke, (C) agreeing that society disapproves of smoking, and (D) having a negative opinion of smoking, by country and Wave (N=57,086 observations from 23,831 respondents). Data are weighted. W=Wave.

|  | **W1 2002** | **W2**  **2003** | **W3 2004** | **W4**  **2005-2006** | **W5**  **2006-2007** | **W6**  **2007-2008** | **W7**  **2008-2009** | **W8**  **2010-2011** | **W9**  **2013-2015** |
| --- | --- | --- | --- | --- | --- | --- | --- | --- | --- |
| **(A) Over half of five closest friends smoke (%)** |  |  |  |  |  |  |  |  |  |
| Canada | 58.87 | 56.93 | 57.64 | 52.19 | 53.00 | 52.24 | 51.62 | 50.07 | 50.54 |
| US | 60.63 | 58.57 | 60.52 | 57.97 | 58.20 | 56.50 | 52.23 | 53.27 | 55.76 |
| UK | 60.59 | 61.39 | 54.86 | 52.77 | 53.92 | 54.67 | 52.37 | 50.78 | 46.87 |
| Australia | 59.71 | 56.91 | 55.35 | 53.53 | 50.38 | 53.50 | 51.65 | 48.43 | 52.18 |
| **Total** | **59.96** | **58.43** | **57.14** | **54.12** | **53.86** | **54.21** | **51.97** | **50.67** | **52.30** |
| **(B) Agree that people important to you believe you should not smoke (%)** |  |  |  |  |  |  |  |  |  |
| Canada | 89.71 | 86.01 | 88.18 | 90.81 | 89.60 | 89.23 | 87.45 | 86.08 | 85.54 |
| US | 89.50 | 88.59 | 89.04 | 90.32 | 91.06 | 88.15 | 86.83 | 87.49 | 75.71 |
| UK | 85.46 | 79.19 | 82.84 | 84.12 | 85.02 | 80.95 | 78.85 | 77.75 | 71.98 |
| Australia | 88.73 | 87.61 | 87.60 | 89.99 | 89.06 | 85.86 | 85.23 | 82.92 | 76.69 |
| **Total** | **88.27** | **85.35** | **86.94** | **88.84** | **88.72** | **86.09** | **84.56** | **83.89** | **77.25** |
| **(C) Agree that society disapproves of smoking (%)** |  |  |  |  |  |  |  |  |  |
| Canada | 88.37 | 88.47 | 89.61 | 91.03 | 90.02 | 91.55 | 88.46 | 85.84 | 80.80 |
| US | 78.93 | 80.66 | 82.80 | 84.53 | 86.22 | 85.99 | 85.66 | 80.90 | 67.76 |
| UK | 77.34 | 80.26 | 83.43 | 85.69 | 86.77 | 85.27 | 83.93 | 82.31 | 75.14 |
| Australia | 81.68 | 82.70 | 87.88 | 87.23 | 88.71 | 88.14 | 83.92 | 80.07 | 78.27 |
| **Total** | **81.51** | **83.08** | **85.91** | **87.14** | **87.94** | **87.77** | **85.54** | **82.34** | **73.78** |
| **(D) Negative opinion of smoking** |  |  |  |  |  |  |  |  |  |
| **Canada (%)** | 62.43 | 56.66 | 54.64 | 57.76 | 56.67 | 56.58 | 54.05 | 48.74 | 58.68 |
| US | 52.75 | 48.19 | 51.10 | 50.58 | 49.92 | 50.03 | 48.49 | 46.35 | 45.18 |
| UK | 47.93 | 44.52 | 45.66 | 51.49 | 47.38 | 46.13 | 44.42 | 40.81 | 45.80 |
| Australia | 55.81 | 51.30 | 52.25 | 53.40 | 54.34 | 51.16 | 53.61 | 50.36 | 52.15 |
| **Total** | **54.59** | **50.24** | **50.93** | **53.33** | **52.12** | **51.02** | **50.09** | **46.80** | **49.37** |

**Supplementary Table 3.** Adjusted associations between all model variables and (A) having over half of five closest friends smoke, (B) agreeing that people important to you believe you should not smoke, (C) agreeing that society disapproves of smoking, and (D) having a negative opinion of smoking, among daily smokers (N=57,086 observations from 23,831 respondents).

|  | **(A) Over half of five closest friends smoke** | |  | **(B) Agree that people important to you believe you should not smoke** | |  | **(C) Agree that society disapproves of smoking** | |  | **(D) Negative opinion of smoking** | |
| --- | --- | --- | --- | --- | --- | --- | --- | --- | --- | --- | --- |
|  | **AOR (95% CI)** | **p** |  | **AOR (95% CI)** | **p** |  | **AOR (95% CI)** | **p** |  | **AOR (95% CI)** | **p** |
| **Wave** |  |  |  |  |  |  |  |  |  |  |  |
| 1 - 2002 (ref) | 1.00 |  |  | 1.00 |  |  | 1.00 |  |  | 1.00 |  |
| 2 - 2003 | 1.04 (0.98-1.10) | .250 |  | **0.71 (0.65-0.78)** | **<.001** |  | 1.03 (0.94-1.12) | .557 |  | **0.86 (0.81-0.91)** | **<.001** |
| 3 - 2004 | 1.03 (0.96-1.10) | .477 |  | **0.77 (0.69-0.86)** | **<.001** |  | **1.22 (1.11-1.35)** | **<.001** |  | **0.88 (0.82-0.94)** | **<.001** |
| 4 - 2005-2006 | 0.98 (0.92-1.06) | .673 |  | 0.90 (0.81-1.01) | .072 |  | **1.31 (1.18-1.47)** | **<.001** |  | 0.97 (0.91-1.04) | .423 |
| 5 - 2006-2007 | 1.00 (0.93-1.08) | .998 |  | 0.92 (0.82-1.04) | .178 |  | **1.38 (1.24-1.55)** | **<.001** |  | **0.93 (0.86-1.00)** | **.045** |
| 6 - 2007-2008 | 1.06 (0.98-1.15) | .137 |  | **0.73 (0.65-0.83)** | **<.001** |  | **1.32 (1.17-1.48)** | **<.001** |  | **0.89 (0.82-0.96)** | **.002** |
| 7 - 2008-2009 | 1.04 (0.95-1.13) | .433 |  | **0.62 (0.55-0.71)** | **<.001** |  | 1.06 (0.94-1.20) | .314 |  | **0.86 (0.79-0.94)** | **.001** |
| 8 - 2010-2011 | 1.02 (0.92-1.13) | .747 |  | **0.67 (0.57-0.78)** | **<.001** |  | 0.89 (0.77-1.03) | .130 |  | **0.83 (0.75-0.91)** | **<.001** |
| 9 - 2013-2015 | 0.95 (0.85-1.07) | .436 |  | **0.54 (0.46-0.64)** | **<.001** |  | **0.74 (0.63-0.87)** | **<.001** |  | 1.04 (0.93-1.17) | .503 |
| **Country** |  |  |  |  |  |  |  |  |  |  |  |
| Canada (ref) | 1.00 |  |  | 1.00 |  |  | 1.00 |  |  | 1.00 |  |
| US | **1.10 (1.03-1.19)** | **.007** |  | 0.99 (0.89-1.10) | .822 |  | **0.63 (0.57-0.69)** | **<.001** |  | **0.71 (0.66-0.77)** | **<.001** |
| UK | 1.03 (0.96-1.11) | .453 |  | **0.58 (0.52-0.64)** | **<.001** |  | **0.59 (0.54-0.65)** | **<.001** |  | **0.66 (0.62-0.71)** | **<.001** |
| Australia | 0.94 (0.87-1.02) | .131 |  | **0.84 (0.75-0.94)** | **.003** |  | **0.74 (0.67-0.82)** | **<.001** |  | **0.88 (0.81-0.95)** | **.001** |
| **Age** |  |  |  |  |  |  |  |  |  |  |  |
| 18-24 (ref) | 1.00 |  |  | 1.00 |  |  | 1.00 |  |  | 1.00 |  |
| 25-39 | **0.54 (0.48-0.60)** | **<.001** |  | **1.15 (1.00-1.33)** | **.045** |  | **1.62 (1.45-1.81)** | **<.001** |  | **1.20 (1.08-1.32)** | **<.001** |
| 40-54 | **0.34 (0.31-0.38)** | **<.001** |  | **1.26 (1.09-1.45)** | **.001** |  | **2.46 (2.20-2.75)** | **<.001** |  | **1.15 (1.04-1.27)** | **.003** |
| 55-max | **0.22 (0.20-0.25)** | **<.001** |  | 1.00 (0.86-1.16) | .954 |  | **2.44 (2.17-2.74)** | **<.001** |  | 0.98 (0.89-1.08) | .766 |
| **Gender** |  |  |  |  |  |  |  |  |  |  |  |
| Female | 1.00 |  |  | 1.00 |  |  | 1.00 |  |  | 1.00 |  |
| Male | **1.05 (1.00-1.11)** | **.041** |  | **0.86 (0.79-0.92)** | **<.001** |  | **0.68 (0.63-0.72)** | **<.001** |  | 1.04 (0.99-1.10) | .094 |

**CONTINUED BELOW.**

HSI=Heaviness of Smoking Index. AOR=Adjusted Odds Ratio, adjusted for all variables in the model. 95% CI=95% confidence interval. 95% CIs are reported to 3 decimal places where they are close to 1.00 (±0.005). Linear, quadratic, and cubic terms for Wave (presented in Table 2 in the manuscript body) are not included here as these terms were added the model as an additional step (see Analyses section).

**Supplementary Table 3 (continued).** Adjusted associations between Wave and country and (A) having over half of five closest friends smoke, (B) agreeing that people important to you believe you should not smoke, (C) agreeing that society disapproves of smoking, and (D) having a negative opinion of smoking, among daily smokers (N=57,086 observations from 23,831 respondents).

|  | **(A) Over half of five closest friends smoke** | |  | **(B) Agree that people important to you believe you should not smoke** | |  | **(C) Agree that society disapproves of smoking** | |  | **(D) Negative opinion of smoking** | |
| --- | --- | --- | --- | --- | --- | --- | --- | --- | --- | --- | --- |
|  | **AOR (95% CI)** | **p** |  | **AOR (95% CI)** | **p** |  | **AOR (95% CI)** | **p** |  | **AOR (95% CI)** | **p** |
| **Ethnicity** |  |  |  |  |  |  |  |  |  |  |  |
| Minority (ref) | 1.00 |  |  | 1.00 |  |  | 1.00 |  |  | 1.00 |  |
| Majority | 1.02 (0.94-1.11) | .557 |  | 0.90 (0.80-1.02) | .115 |  | **1.48 (1.35-1.62)** | **<.001** |  | 0.99 (0.91-1.07) | .817 |
| **Income** |  |  |  |  |  |  |  |  |  |  |  |
| Low (ref) | 1.00 |  |  | 1.00 |  |  | 1.00 |  |  | 1.00 |  |
| Moderate | **0.89 (0.84-0.95)** | **<.001** |  | **1.25 (1.15-1.37)** | **<.001** |  | **1.31 (1.21-1.42)** | **<.001** |  | 1.00 (0.94-1.07) | .761 |
| High | **0.69 (0.64-0.73)** | **<.001** |  | **1.50 (1.36-1.65)** | **<.001** |  | **1.43 (1.31-1.56)** | **<.001** |  | **1.07 (1.00-1.15)** | **.028** |
| No answer | **0.86 (0.78-0.95)** | **.004** |  | 1.04 (0.91-1.20) | .513 |  | 0.89 (0.79-1.00) | .072 |  | **0.80 (0.72-0.88)** | **<.001** |
| **Education** |  |  |  |  |  |  |  |  |  |  |  |
| Low (ref) | 1.00 |  |  | 1.00 |  |  | 1.00 |  |  | 1.00 |  |
| Moderate | **0.81 (0.76-0.86)** | **<.001** |  | 1.01 (0.93-1.10) | .673 |  | **1.10 (1.02-1.19)** | **.007** |  | **1.18 (1.11-1.25)** | **<.001** |
| High | **0.57 (0.53-0.62)** | **<.001** |  | **1.14 (1.02-1.28)** | **.018** |  | **1.37 (1.23-1.52)** | **<.001** |  | **1.41 (1.30-1.52)** | **<.001** |
| No answer | 1.08 (0.73-1.62) | .677 |  | 0.88 (0.50-1.54) | .664 |  | **0.61 (0.39-0.94)** | **.026** |  | 1.79 (1.12-2.85) | .013 |
| **HSI** | **1.10 (1.09-1.12)** | **<.001** |  | **0.94 (0.92-0.96)** | **<.001** |  | 1.01 (0.99-1.03) | .251 |  | **0.96 (0.95-0.98)** | **<.001** |
| **Mode** |  |  |  |  |  |  |  |  |  |  |  |
| Telephone (ref) | 1.00 |  |  | 1.00 |  |  | 1.00 |  |  | 1.00 |  |
| Internet | 1.03 (0.93-1.14) | .556 |  | **0.55 (0.48-0.63)** | **<.001** |  | **0.63 (0.55-0.72)** | **<.001** |  | **0.76 (0.69-0.84)** | **<.001** |
| **Time-in-sample** | **0.93 (0.91-0.95)** | **<.001** |  | 1.00 (0.98-1.03) | .512 |  | **1.05 (1.02-1.08)** | **<.001** |  | 0.99 (0.97-1.01) | .810 |
| **Time-between-Waves** | **1.05 (1.00-1.10)** | **.040** |  | **1.11 (1.04-1.19)** | **.001** |  | 0.95 (0.89-1.02) | .215 |  | **0.94 (0.90-0.99)** | **.018** |

HSI=Heaviness of Smoking Index. AOR=Adjusted Odds Ratio, adjusted for all variables in the model. 95% CI=95% confidence interval. 95% CIs are reported to 3 decimal places where they are close to 1.00 (±0.005). Linear, quadratic, and cubic terms for Wave (presented in Table 2 in the manuscript body) are not included here as these terms were added to the model as an additional step (see Analyses section).


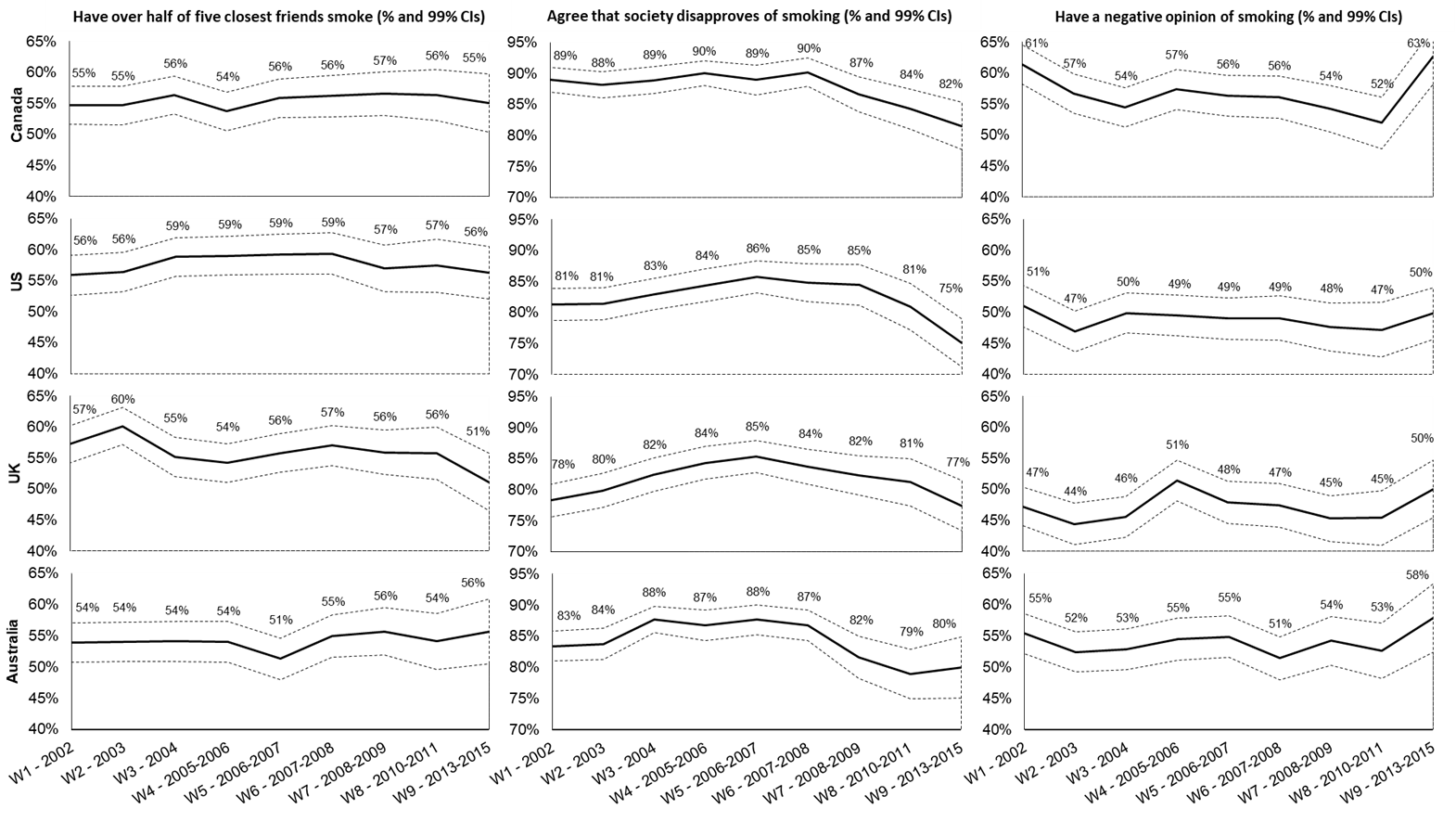
**Supplementary Figure 1.** Average predicted probabilities of having over half of five closest friends smoke, agreeing that society disapproves of smoking, and having a negative opinion of smoking, by Wave and country (N=57,086 observations from 23,831 respondents). Average predicted probabilities and 99% confidence intervals (CIs) are generated from the Wave*Country interaction terms in the binary logistic regression analyses, adjusted for age, gender, ethnicity, income, education, heaviness of smoking, survey mode, time in sample, and time between Waves. Data are weighted. 99% CI=99% confidence interval. W=Wave.
